# Effects of K-wire diameter and insertion angle on femoral bone medial closing-wedge osteotomies: a finite element study

**DOI:** 10.1038/s41598-025-04260-5

**Published:** 2025-06-20

**Authors:** Ayda K. Dastgerdi, Alireza Y. Bavil, Markus T. Berninger, Imke A. K. Fiedler, Björn Busse, Matthias Krause, Felix N. von Brackel

**Affiliations:** 1 Department of Osteology and Biomechanics, University Medical Center Hamburg-Eppendorf, Hamburg, Germany; 2 Australian Centre for Precision Health and Technology (PRECISE), Griffith University, Gold Coast, QLD Australia; 3 Interdisciplinary Competence Center for Interface Research (ICCIR), University Medical Center Hamburg-Eppendorf, Hamburg, Germany; 4 Department of Trauma, Hand and Reconstructive Surgery, University Medical Center Hamburg-Eppendorf, Hamburg, Germany

**Keywords:** K-wire, Biomechanics, In silico, Hinge fracture prevention, Stress and strain analysis, Surgical optimization, Structural stability, Orthopedics, Digital twin, Biomedical engineering, Bone

## Abstract

Medial closing-wedge surgery for distal femoral osteotomy is employed to correct genu valgum by correcting coronal plane malalignment. This procedure involves pre-surgery planning, creating a wedge incision, performing the osteotomy, and stabilizing with plates and screws. However, hinge fractures during wedge closure present significant challenges, often necessitating revisions. Contemporary solutions have explored the use of k-wires, and this study investigates their biomechanical implications. The interplay between k-wire insertion angle and diameter, often overlooked in existing literature, is a critical determinant of their efficacy in achieving successful osteotomies, highlighting gaps in our understanding of these key parameters. We hypothesize that k-wire mechanics vary with insertion angle and diameter. This study examines the introduction of k-wires at different angles (30°, 45°, and 60°) and diameters (1.6, 1.8, and 2 mm) using computed tomography-based finite element models to assess structural integrity during femoral medial closing-wedge osteotomy. Results reveal angle-dependent stress variations, with 60° configurations exhibiting favorable patterns that reduce tensile and compressive loads and plastic deformation—crucial in preventing hinge fractures. Diameter variations show no significant differences in stresses or system stiffness. It was also found that while angle significantly affects stresses, lower diameters appear optimal only in combination with higher angles. Comparative analysis of k-wire systems with a naïve model demonstrates that k-wires at a 60° angle reduce tensile and compressive loadings and plastically deformed volume fractions, thus lowering fracture risk. This study underscores the importance of optimizing k-wire placement and configuration, particularly highlighting the significance of the insertion angle. Future research should expand the range of angles and diameters tested and examine different femoral geometries and osteotomy angles to provide a more comprehensive understanding and enhanced clinical application.

## Introduction

**Fig. 1:**
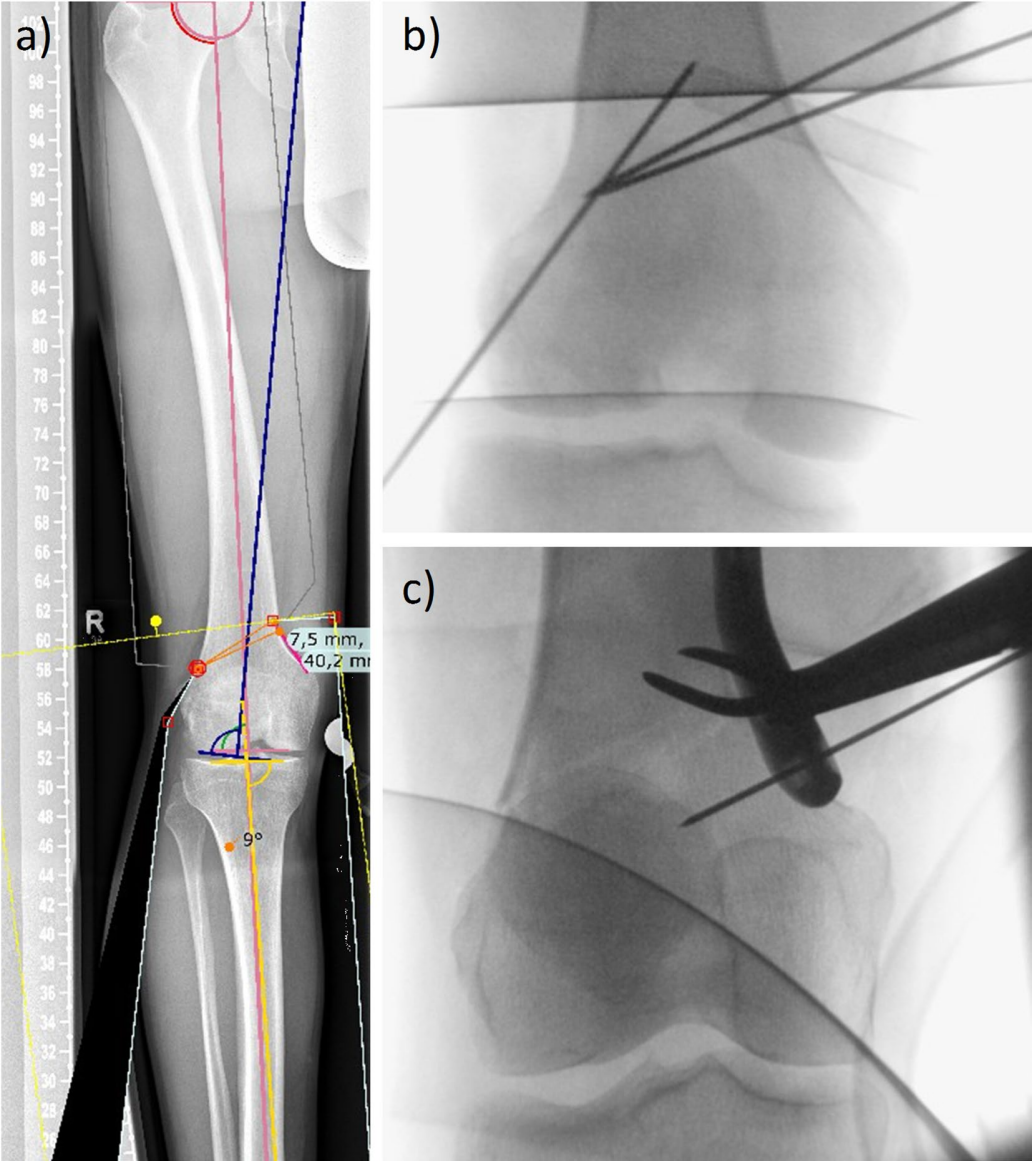
Planning and intraoperative imaging. Knee with a valgus alignment and planning for the surgery indicating the wedge for an exemplary patient (**a**). Intraoperatively, wedge location is planned (**b**). Closing of the wedge may in some cases lead to a fracture at the hinge (**c**), which may be prevented by insertion of a Kirschner wire (k-wire) to protect the hinge.

Medial closing-wedge surgery for distal femoral osteotomy is a surgical solution aimed at rectifying genu valgum (Fig. 1a), a condition characterized by an inward angulation of the knee. This surgery is indicated for patients experiencing lateral knee joint osteoarthritis to maintain a functioning knee by distributing the stresses to the medial knee joint compartments^1^. This procedure requires patient evaluation and accurate surgical planning (Fig. 1a) for a precise wedge incision in the distal femur^2,3^. During the surgery, a controlled bone cut (i.e., osteotomy) is performed on the distal femur, creating a wedge-shaped gap on the medial side (Fig. 1b). This gap is then closed by bowing the remaining bone at the hinge of the wedge to close the introduced gap. Stabilization of the reconstructed bone is achieved through fixation using plates and screws, ensuring proper bone healing^4^.

However, during wedge closure the remaining bone may fracture at the hinge (Fig. 1c). This leads to pronounced instability, which makes further surgical treatment necessary by means of contralateral fixation. This is associated with significantly more exogenous material in the body, an increased risk of infection, and thus complications, and increased instability^5,6^. Nonetheless, the treatment is necessary to maintain the healing of the completely detached femur and to enable the patient to regain functional independence without inducing pseudarthrosis due to high micromotion at the bone gap^7–9^.

While medial closing-wedge surgery is generally effective in correcting genu valgum and improving patient symptoms, challenges such as a hinge fracture exist. In response, Kirschner wires (k-wires) are currently being discussed as an innovative solution to such fractures^10^. K-wires, with their inherent versatility, present a promising approach for enhancing the efficacy of osteotomies during closing-wedge surgery by preventing hinge fracture through stress shielding^11^. Inserting a k-wire is believed to reduce hinge stress and fracture risk by reallocating forces during wedge closure. The effectiveness of k-wires in orthopedic procedures, particularly in the context of closing-wedge surgery, remains a subject of great clinical interest. The interplay between k-wire insertion angle and diameter, often overlooked in existing literature, is a critical determinant of their efficacy in achieving successful osteotomies, highlighting gaps in our understanding of these key parameters. In silico methods such as finite element (FE) modeling can be exploited to investigate the interplay of these parameters^12–15^. The FE method has shown robust predictability in the field of orthopedic biomechanics; therefore it is a strong tool to analyze scenarios that are otherwise impossible or resource intensive^16–18^.

However, there exists a notable gap in the literature, as no study has systematically examined the collective influence of these two critical parameters on the mechanics of the system during wedge closure. For the proper selection of k-wire diameter and insertion angle, a thorough evaluation of key mechanical aspects across various scenarios is essential.

This investigation aims to address this research gap by examining the mechanical implications associated with different k-wire diameters and insertion angles, specifically focusing on their combined effects during wedge closure. Using computed tomography (CT)—based FE analysis, this study integrates simulated surgical scenarios with varying k-wire angles (30°, 45°, and 60°) and diameters (1.6mm, 1.8mm, and 2mm) to statistically elucidate their individual and combined effects. Moreover, a comparative analysis is conducted between the performance of k-wire FE models and a naïve (i.e., without k-wire) FE model to discern potential disparities between the two systems. We hypothesize that the mechanical performance of k-wires can be influenced by alterations in both insertion angle and implant diameter.

## Material and methods

### Imaging and CAD model development

**Fig. 2:**
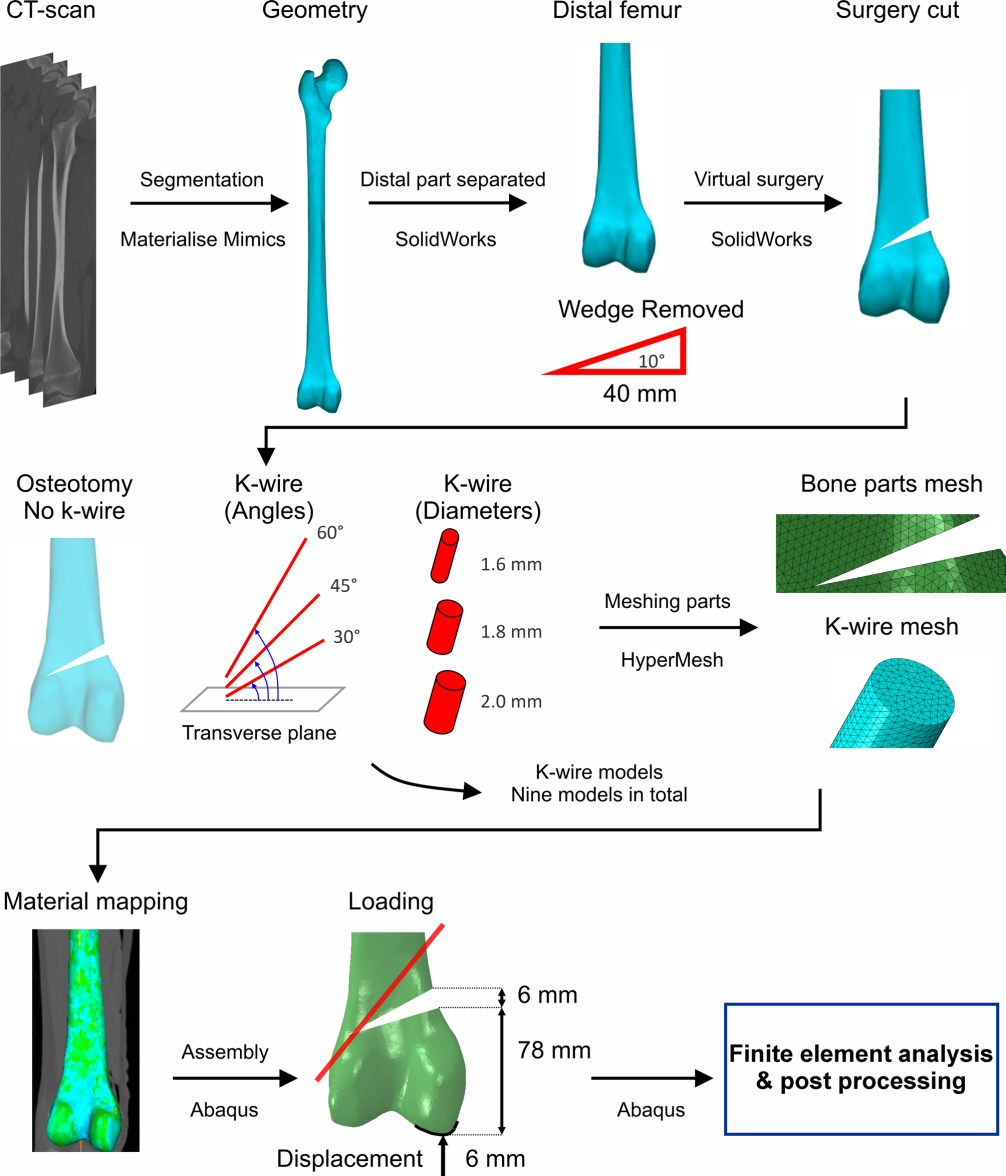
Overview of the workflow used in this study. The segmentation of bone geometry from CT data and introduction of an exemplary surgical wedge (first row), placement of k-wire in different configurations and meshing the system instances (second row) including both without k-wire and with k-wire, assigning subject-specific material properties and running the finite element analysis (third row).

CT imaging of the right knee of a 46-year-old female, deceased without metabolic bone diseases, was performed using an X-ray tube current of 297mA and a peak voltage of 120kV with a spatial resolution of 0.33mm and a slice thickness of 0.8mm. The acquired raw DICOM data from the CT scan were subsequently processed using Mimics software (Version 21.0, Materialise) to facilitate segmentation and the generation of a three-dimensional (3D) model of the femur. Following this, the constructed model underwent further refinement using 3-Matics software (Version 12.0, Materialise) to eliminate surface irregularities and noise (Fig. 2).

The medial closing-wedge osteotomy of the femur was defined by two experienced surgeons and executed using SolidWorks (Version 2019, Dassault Systèmes, Vélizy-Villacoublay, France). The osteotomy plane was positioned on the medial side with a 10° angle, approximately 78 mm proximal to the distal femoral articular surface, corresponding to the supracondylar region commonly targeted in distal femoral osteotomies (Fig. 2). The osteotomy procedure commenced in the lateral supracondylar region and extended towards the medial femoral condyle, ensuring continuous contact between the proximal and distal femur segments on the medial side. Furthermore, a computer-aided design (CAD) model of the k-wire was created using SolidWorks, incorporating three distinct diameters: 1.6 mm, 1.8 mm, and 2 mm. To assemble the k-wire to the bone, three different angles between the k-wire and the transverse plane were considered, specifically 30 degrees (30°), 45 degrees (45°), and 60 degrees (60°) (Fig. 2). The selected angles were informed by previous work by Erkan et al.^19^, which demonstrated that bicortical k-wire engagement requires a minimum insertion angle of 30°, with 45° and higher angles providing more reliable fixation outcomes. The upper bound of 60° was set by surgical anatomy; at steeper trajectories, the required lateral entry point becomes obstructed by soft-tissue structures (e.g., iliotibial band, biceps femoris, neurovascular bundle), making wire insertion impractical under standard fluoroscopic guidance. This range allowed us to evaluate k-wire orientation within both biomechanically relevant and anatomically feasible limits. The combination of these three k-wire diameters with the three positioning angles resulted in a total of nine distinct models.

### Meshing and material assignment

The constructed components were imported into HyperMesh software (Altair, Frisco, TX, USA) to create volumetric mesh for the FEA. The convergence study led to an average mesh size of 2 mm for the bone and 0.2 mm for the k-wires and the k-wire and bone contact surface on the femur. This led to the creation of an intricately detailed femur model, comprising 470,000 linear tetrahedral (C3D4) elements. In-house analyses confirmed that the C3D4 element type exhibited comparable performance to other element types and was chosen for its computational efficiency.

The femur model’s material properties were determined based on the gray values from individual CT scans, employing established material mapping equations^20,21^ allowing for heterogeneous, subject-specific mechanical representation (see Eqs. (1) and (2)). Ten distinct material properties were assigned to the femur volume mesh based on CT gray values^22^.

1 $${\rho }_{app}=0.001029GV+0.114259 g/{cm}^{3}$$

2 $$E = 6850\rho_{app}^{1.49} MPa$$

where, ρ_app_ is the apparent bone density, GV is the assigned mesh grayscale value, and E is the Young’s modulus.

The k-wires were characterized as medical grade titanium with isotropic linear elastic–plastic materials, with a Young’s modulus of 110 GPa, Poisson’s ratio of 0.3, and a yield stress of 1240 MPa^23^. Subsequently, the meshed models were imported into Abaqus (SIMULIA, Paris, France) for the execution of FE analyses.

### Loading and boundary conditions

Uniform boundary conditions were imposed across all FE models. The superior surface of the proximal femoral segment was constrained in all spatial directions for every configuration. To achieve wedge closure, a total displacement of 6 mm was applied on the reference point coupled to the inferior surface of the distal femoral segment. This value corresponds to closing a 10° wedge at the osteotomy level and reproduces the intra-operative maneuver when the hinge is most at risk, making 6-mm displacement control the clinically relevant loading mode. Additionally, preliminary analysis showed that a 6 mm displacement is sufficient for full closure of the introduced wedge in the FE models. Interactions between the superior and inferior wedge surfaces occurring on proximal and distal bone fragments were modelled using tangential surface-to-surface contact, featuring a friction coefficient of 0.3 (Fig. 2)^24,25^. Furthermore, a hard contact pressure-clearance relation was defined to prevent any interpenetration of the bone segments during the wedge closure process. Two surface-to-surface contacts were established between the two ends of the k-wire and the proximal and distal femoral segments, respectively. Additionally, a frictionless surface-to-surface contact between the outer surface of the k-wire and the inner surface of the marrow canal was defined. These conditions were consistently applied across all models to ensure uniformity and comparability in the simulations and subsequent results of the study.

### Data analysis and statistics

Compressive and tensile maximum principal stresses on the site of the hinge along with the volume fraction of elements experiencing plastic deformation on the distal bone instance, were quantified for the distal femoral segment to assess potential failure risks across various models. The number of elements experiencing *von Misses* stresses of greater than 133 MPa divided by the total number of elements in the distal part of the bone was defined as the volume fraction. In addition, longitudinal stress in the direction of the main axis of the femur was measured along a predefined trajectory originating from the lateral side and extending towards the medial side of the hinge on the distal femur. This is of particular importance, since bone responds differently to tensile and compressive stresses^26^. Moreover, the overall stiffness of the models was computed to evaluate their structural stability.

For statistical comparison of either diameter or angle, model results for the same angle or the same diameter were pooled, respectively. ANOVA with subsequent *post-hoc* testing with Bonferroni correction was performed for multiple comparisons. A significance level of *p* < 0.05 was defined to ascertain statistical significance. In order to present the output measures effectively, two-dimensional heatmaps were utilized to depict the measures across the range of angles and diameters.

In order to assess the performance disparity between k-wire FE models and a naïve FE model, relative error was computed across four key output measures: maximum tensile and compressive stresses, stiffness, and volume fraction of elements experiencing plastic deformation in the distal bone instance. The relative error values were subsequently color-coded for visual clarity. The green spectrum represents lower magnitudes of output measures in the k-wire model compared to the naïve model, while the red spectrum indicates higher magnitudes of these measures in the k-wire model. Darker shades within each spectrum denote a greater difference between the k-wire and naïve models. This color-coding scheme facilitates an intuitive comparison of the performance disparities between the k-wire and naïve FE models.

## Results

### Angle variation

**Fig. 3:**
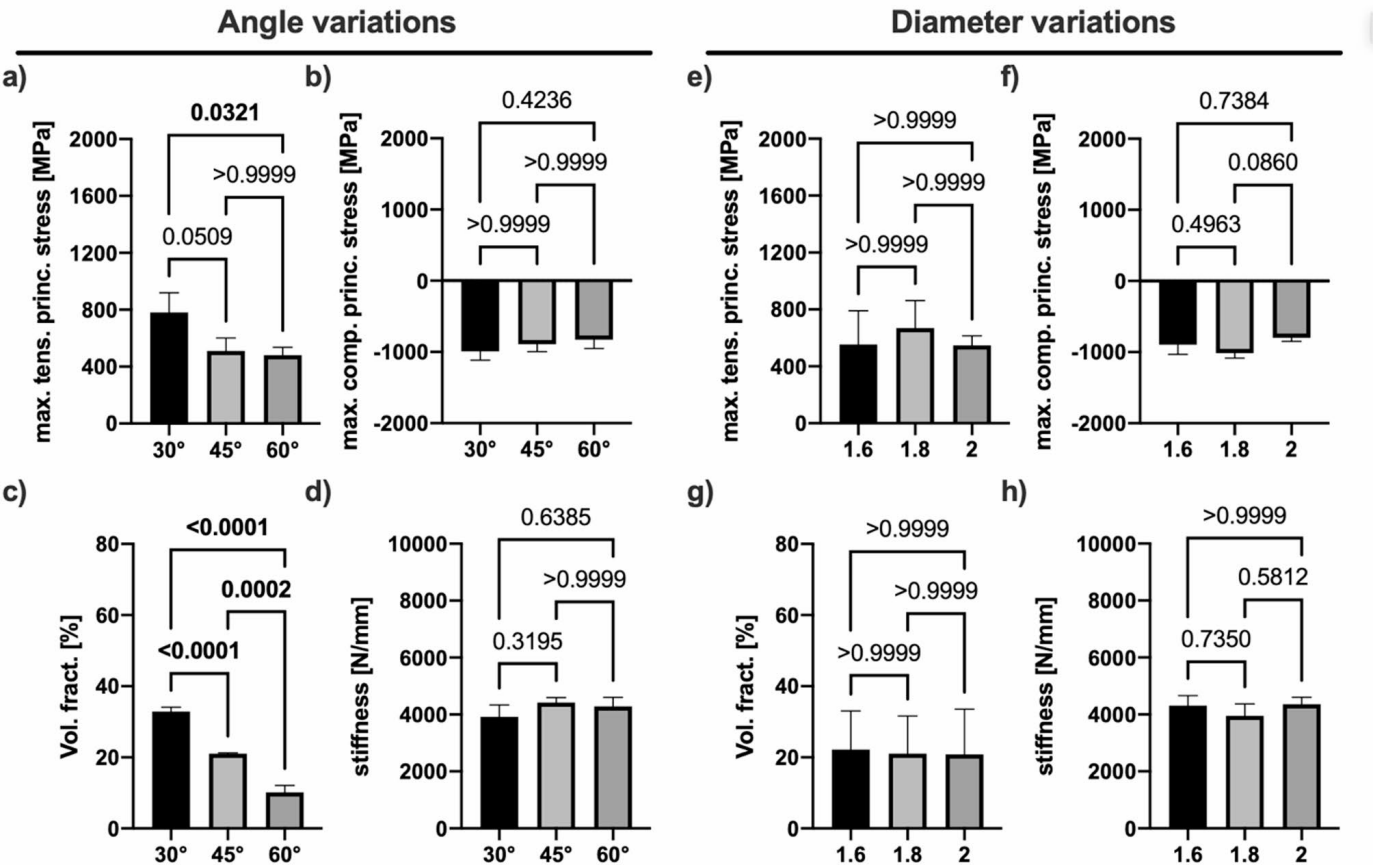
Output measures versus angle variation (left) and diameter variation (right) of a defined trajectory at the hinge bone. Differences were detected for variation in the angle of insertion of the k-wire for maximum tensile principal stresses, with 30° exhibiting the highest and 60° the lowest stresses (**a**). No differences were measured for compressive stress (**b**). The volume fraction of plastically deformed elements was lowest at a 60° insertion angle, with a stepwise increase as the angle decreased (**c**) while no difference in stiffness was measured for the assembly (**d**). For variations in diameter, no significant differences were measured for all parameters (**e**–**h**).

Peak principal stresses in the distal femur region showed measurable variability depending on the angle of insertion of the k-wire (Fig. 3a, d). In general, the 60° group resulted in the lowest maximum principal stress on the distal part of the femur, followed by the 45° and 30° groups (Fig. 3a, b). Significant differences were observed between 30° and 60° in maximum tensile principal stresses, and a stepwise trend towards lower maximum compressive principal stresses was observed from 30°, 45° to 60°. The proportion of elements undergoing plastic deformation in the distal section of the femur was significantly decreased by the increase in the angle of the k-wire. The highest fraction of plastically deformed elements was measured for 30°, and the lowest for the case of 60° (p < 0.005 for all comparisons; confidence intervals are reported in Suppl. Table S1) (Fig. 3c). No significant differences were observed when quantifying the stiffness depending on the angle variation (Fig. 3d).

### Diameter variation

Diameters of 1.6, 1.8, and 2.0mm did not show significant differences between the three groups for tensile (Fig. 3e) and compressive (Fig. 3f) stresses. No significant differences were observed between the three diameters for the volume fraction of elements undergoing plastic deformation (Fig. 3g). When varying the diameter and quantifying the stiffness, no significant differences were observed among the different diameter groups (Fig. 3h).

### Parameter combinations

**Fig. 4:**
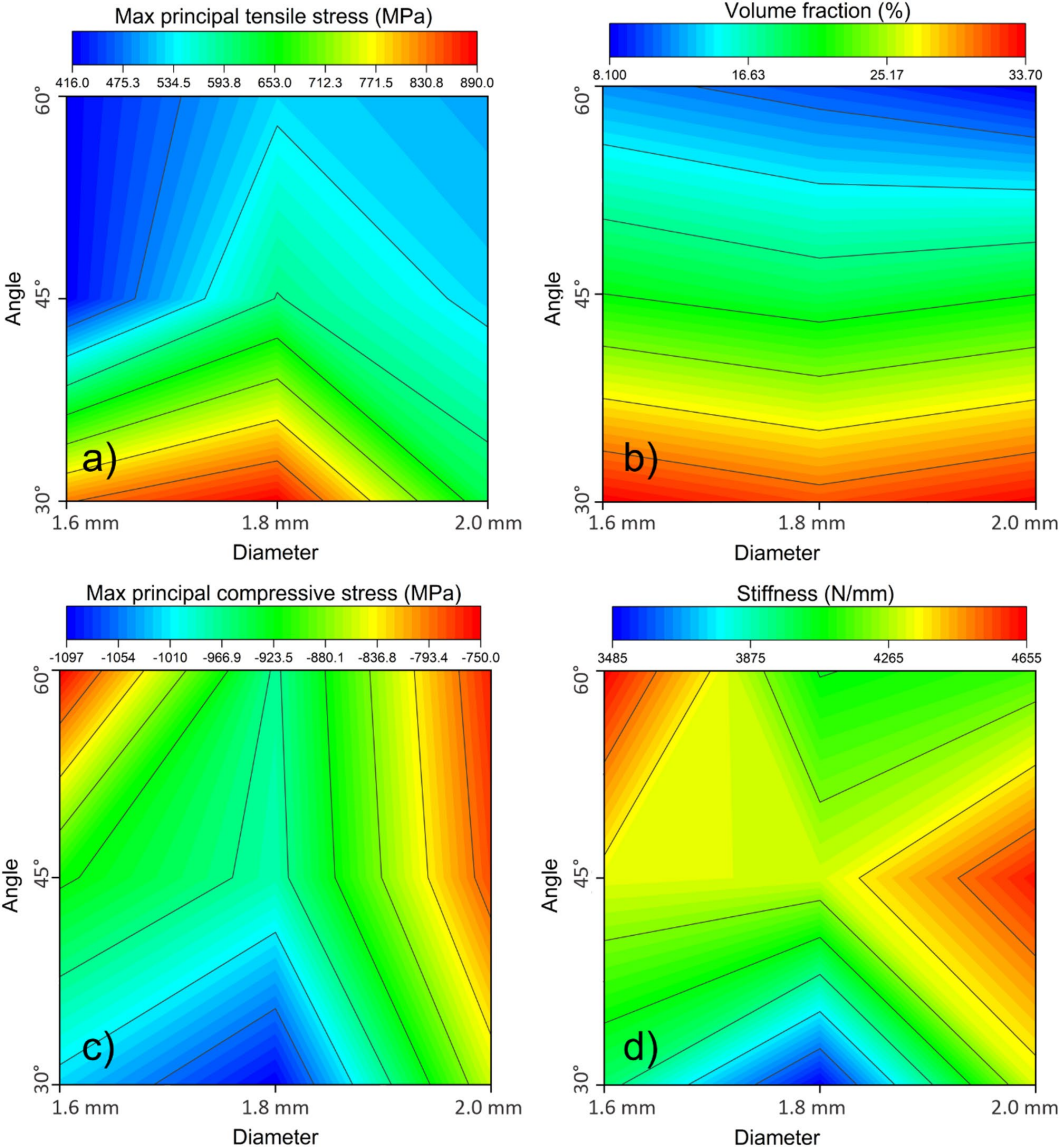
Heatmaps indicating the output measure combinations for angle and diameter variations: Angle-diameter heatmap for tensile maximum principal stresses (**a**), compressive maximum principal stresses (**b**), volume fraction (**c**), and stiffness (**d**).

Combined effects of k-wire angles and diameters are visualized using heatmaps. Maximum principal tensile stresses was observed for the angle of 60° and a diameter of 1.6mm (Fig. 4a). Compressive stress exhibits the lowest values at 60° with similar values for 1.6 mm and 2.0 mm diameter (Fig. 4b). The volume fraction of plastically deformed elements was lowest for any diameter at an angle of 60°, with an almost linear decrease along the angle variation, which indicates no variability depending on the diameter (Fig. 4c). Among the tested configurations, 60°exhibited the highest stiffness, leading to increased overall system stability. Expanding on the diameter parameter, within the 30° group, the largest diameter (i.e., d = 2mm) resulted in the highest stiffness. Similarly, for the 45° group, the highest diameter showed the highest stiffness. In contrast, within the 60° group, the smallest diameter (i.e., d = 1.6) resulted in the highest stiffness (Fig. 4d).

### Longitudinal normal stress on distal site

**Fig. 5:**
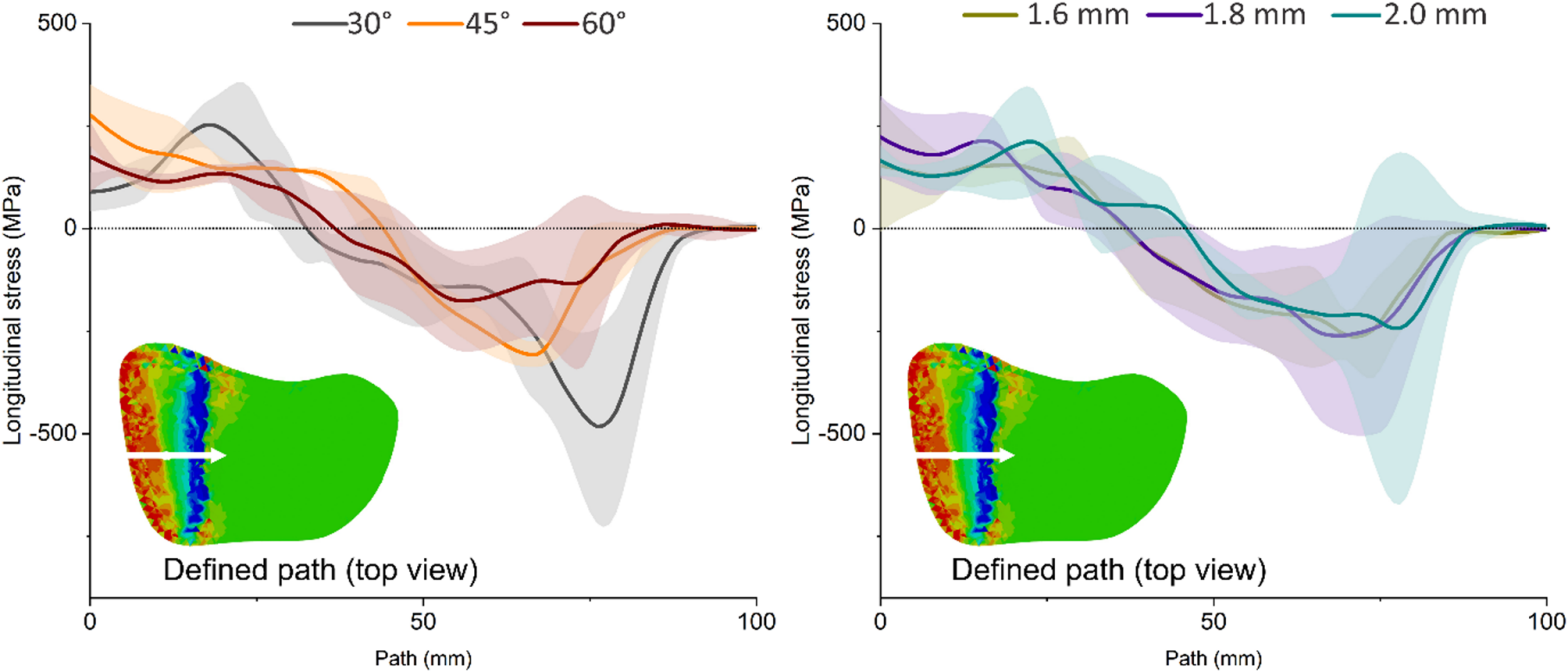
Longitudinal normal stress induced on the superior surface of distal femur in contact with the proximal part for different angles (left) and diameters of the k-wire (right). Positive values indicate tensile and negative values indicate compressive stresses. The path on which the values have been read is highlighted using the white arrows, running on the superior osteotomy surface from the lateral cortico-osteotomy junction traversing medially toward the medial hinge apex, aligned with the expected line of stress transfer during wedge closure.

The results of longitudinal normal stresses along a defined path—originating from the lateral side and extending towards the medial side of the hinge on the distal femur—at the contact area between distal and the proximal parts of the femur is depicted in Fig. 5. A 30° k-wire assembly resulted in the highest magnitude of normal stresses at the contact area followed by 45° and 60°, indicating that 60° to introduce the lowest longitudinal stresses to the remaining bone at the tip of the wedges. Changes in the diameters did not result in a discernible variation in the longitudinal stresses along the defined path.

### K-wire finite element models vs. naïve finite element model

The comparison between the k-wires FE models and the naïve model yielded important insights summarized in Table 1. In terms of maximum principal stress, k-wires angled at 60° exhibited lower tensile stress levels in the femur compared to the naïve model. However, k-wires angled at 30° across all diameters, resulted in higher tensile stresses compared to the naïve model in the same region. Conversely, all k-wire models showed higher maximum compressive stress levels than the naïve model. For k-wires angled at 60° with diameters of 1.6mm and 2mm, the increase in compressive stress was less than 10% compared to the naïve model. Regarding stiffness, the naïve model demonstrated higher stiffness compared to the k-wire models across all angles and diameters. Concerning volume fraction, only k-wires angled at 60°, regardless of the diameter, showed a lower volume fraction of plastically deformed elements compared to the naïve model. Overall, the findings suggest that k-wires angled at 60°, especially with diameters of 1.6mm and 2mm, exhibit superior performance in tension and volume fraction when compared to the naïve model.

**Table 1:**
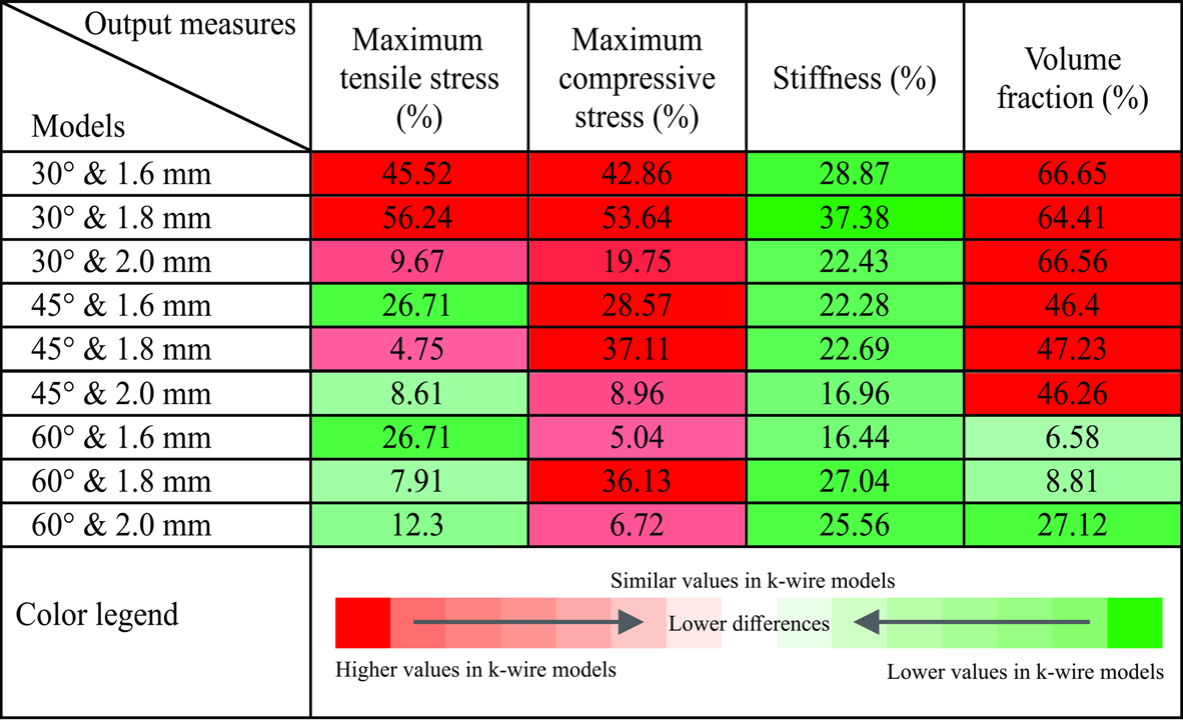
Relative error (%) comparison between k-wire finite element (FE) models and the naïve FE model across four output measures: maximum tensile and compressive stresses, stiffness, and volume fraction of elements experiencing plastic deformation in the distal bone instance.

The red spectrum indicates higher output values in the k-wire model compared to the naïve model, while the green spectrum indicates lower output values in the k-wire FE model compared to the naïve model. Darker shades represent greater relative differences between the naïve and k-wire models.

## Discussion

A wedge osteotomy is performed to surgically correct a malalignment of the leg axis, such as unilateral massive joint degeneration^27^. Depending on whether the procedure is performed on the medial or lateral side, the wedge can correct a varus or valgus alignment^28,29^. Closing the wedge is challenging because bringing the proximal and distal bone segments together, which are still rigidly connected at the tip of the wedge (the so-called “hinge”), makes them susceptible to fracture due to the significant deformation that the remaining bone must undergo. To overcome this problem, k-wires have been proposed^30^. Here we investigated the introduction of k-wires at different angles (30°, 45°, and 60°) and diameters (1.6, 1.8, and 2) using FE models to assess structural integrity during wedge closure. The stiffness results indicated that k-wire configurations with an angle of 30° demonstrated lower stability compared to angles of 60° and 45°. The results of this study were consistent with the hypothesis, indicating that the mechanics of the k-wire system are affected by the changes made in the insertion angle and the diameter of the implant while the k-wire insertion angle is the more dominant parameter.

Examining the influence of different insertion angles of the k-wire reveals a dedicated mechanical relation indicating angle-dependent system responses. Increased parameters of stress and plastic volume deformation are indicators of high mechanical loading making the bone more prone to fractures. Therefore, the aim of introducing k-wires is to reduce maximum stresses on the bone to prevent bone tissue fractures during the process of closing the wedge. Here, especially 60° exhibits a favorable mechanical pattern with a reduction of the tensile and compressive stresses as well as reduced plastically deformed volume fraction, lowering the risk of a fracture during wedge closure and potentially preventing revisions. This effect may be caused by the k-wire undergoing more compressive loading in the 60° setup, rather than more bending-type loading occured at 30°, thus more loads may be transferred by a 60° assembly from cortex to cortex while 30° mostly induces a bending of the k-wire. Aligned with the compressive load vector, the 60° wire behaves as an axial strut, channeling closing forces directly between cortices and reducing hinge bending. In contrast, shallower orientations force the wire into bending, shift load back onto the bone, and elevate local stress and plastic deformation. Furthermore, the lower volume fraction of elements undergoing plastic deformation at 60° indicates a lower risk of bone tissue yielding during wedge closure. However, a steeper k-wire angle does not significantly affect the stiffness of the full assembly. Global stiffness is dictated mainly by the intact cortical bridge at the hinge, whose cross-sectional area far exceeds that of the k-wire. Altering wire orientation from 30° to 60° redistributes local stresses—lowering tensile peaks and plastic deformation—but adds little axial rigidity to influence the overall force–displacement response. This, in turn, means a similar effort is required to close the wedge during surgery for all three angels tested. Steeper angles, i.e., 60,° induce less shear and reduce the bending of the k-wire, fostering a more effective compressive load transfer. This shift of loading regime aligns with the bone’s inherent anisotropic material properties designed mainly to resist compressive loadings, rather than transverse^31–33^. This tension–compression pattern across the hinge is consistent with expected biomechanical behavior during wedge closure, with tensile forces dominating laterally and compressive forces medially. Such loading distribution plays a critical role in fracture susceptibility^34^, and the ability of the k-wire to mitigate these forces, particularly at steeper insertion angles, is a key factor in its mechanical performance.

Considering the k-wire diameters, no statistically significant differences were observed between the output parameters among the diameters measured. Notably, an increase in k-wire diameter does not lead to a significant change in system stiffness. Interestingly, the 1.8 mm configuration showed both higher principal stresses and lower stiffness than the 1.6 mm configuration. This may reflect a biomechanical trade-off: while the 1.8 mm wire causes more local bone removal than the 1.6 mm, it does not offer sufficient cross-sectional stiffness to compensate through effective load redistribution. In contrast, the 2.0 mm wire, despite causing even more bone removal, contributes enough structural rigidity to reduce stress in the adjacent bone and increase global system stiffness. While these differences were not statistically significant, the results suggest that intermediate diameters may underperform when the balance between structural support and bone preservation is suboptimal. This phenomenon, together with the limited range of diameters tested, may help explain the lack of consistent trends in the diameter comparison. Examining the combined effect of angle and diameter using heatmapping (Fig. 4) suggests that higher angles and lower diameters may constitute an optimal configuration, with the 1.6 mm wire at 60° displaying the lowest tensile stress and plastically deformed volume. Further analyses involving additional femurs and a broader diameter range may be required to detect diameter-related trends that may not emerge in the present single-specimen dataset. Therefore, the mechanical loading of the bone appears to be more sensitive to changes in angle, emphasizing its importance compared to k-wire diameter.

The comparative analysis between the k-wire FE models and the naïve FE model revealed significant variations. Notably, k-wires angled at 60° exhibited reduced tensile stress levels and plastically deformed elements’ volume fractions compared to the naïve model. Other configurations predominantly resulted in increased stresses and yielded elements’ volume fractions (see Table 1). Moreover, all k-wire models displayed higher compressive stress levels than the naïve model. Additionally, the naïve model consistently demonstrated greater stiffness than the k-wire models across various angles and diameters. Overall, while k-wires may reduce the risk of system failure by diminishing maximum tensile stress and volume fraction, careful optimization of k-wire placement and configuration is essential to minimize potential adverse mechanical outcomes.

This study is subjected to a few limitations. We have examined three increments for angle and diameter only, which may be expanded to create a direct function derived from the data. Although the absence of bone phantom calibration may limit the accuracy of absolute material properties, the study design, varying only k-wire angle and diameter under consistent conditions, ensures the validity of comparative mechanical trends across models. Furthermore, this study focuses on a single wedge geometry without accounting for variations in osteotomy location relative to anatomical landmarks such as the joint line, which may differ due to surgical decisions or intraoperative deviations. The present models assume a frictionless bone–k-wire interface; potential effects of bone-wire friction or bonding were not investigated. The model also represents a best-case scenario with ideal execution, which may not fully capture the variability present in clinical practice. Additionally, this study lacks experimental validation. While finite element analysis is an accepted tool, supporting the numerical findings with in vitro or in vivo data would strengthen their clinical relevance. Lastly, we have calculated nine models and a naïve model based on the CT data of a single patient, which may limit the generalizability of the findings to other subjects with different underlying pathologies. These limitations call for future investigation of different femoral geometries and different osteotomy angles to provide a more comprehensive understanding and wider clinical application of the findings.

Although the 60° k-wire configuration demonstrated superior biomechanical performance, achieving this steep trajectory intraoperatively can be challenging due to patient anatomy, surgical exposure, and instrumentation constraints; surgeons must therefore balance mechanical benefit with procedural feasibility, potentially using patient-specific 3D planning or intraoperative navigation to guide placement. Future work can extend the present quasi-static wedge-closure model to (i) include plate-and-screw fixation with cyclic gait loading and (ii) examine patient-specific variations—such as varus/valgus alignment and osteoporotic bone quality—using multiple femoral geometries and musculoskeletal force inputs. Simulation of the healing process after osteotomy would also add an additional layer of insights in future studies. Additionally, future work should parametrically vary the bone wire friction coefficient or simulate press fit conditions to quantify their impact on load transfer along the wire.

In conclusion, the integration of k-wires at specific angles and diameters showed promising potential for improving the results of wedge closure in corrective leg axis surgery. Here, we have identified the angle as the most important for optimal configurations, with 60° favored over 45° and 30°, and diameter playing a lesser role compared to angle. The addition of k-wire to the surgical procedure may reduce the susceptibility to hinge fractures during wedge closure, especially when using a 60° supporting k-wire for leg axis corrections.

## Supplementary Information

Supplementary Information.

## Data Availability

The datasets used and/or analyzed during the current study available from the corresponding author on reasonable request.
