# Supplementary material for: Effects of K-wire diameter and insertion angle on femoral bone medial closing-wedge osteotomies: a finite element study
Source: Sci Rep. 2025 Jun 20;15:20116. doi: 10.1038/s41598-025-04260-5 (PMC12181366; doi:10.1038/s41598-025-04260-5)
Supplement: Supplementary file 1 — Supplementary Information. [file 41598_2025_4260_MOESM1_ESM.pdf]

Effects of K-wire diameter and insertion angle on femoral bone medial closing-wedge osteotomies: a finite element study

(Supplementary material)

Ayda K. Dastgerdi[1, 2, 3, §], Alireza Y. Bavil[1, 2, 3, §], Markus T. Berninger[4], Imke A. K. Fiedler[1,3], Björn Busse[1, 3], Matthias Krause[4] and Felix N. von Brackel[1, 3*]

[1]Department of Osteology and Biomechanics, University Medical Center Hamburg-Eppendorf, Hamburg, Germany

[2] Australian Centre for Precision Health and Technology (PRECISE), Griffith University, Gold Coast, QLD, Australia, Griffith University, QLD, Australia

[3]Interdisciplinary Competence Center for Interface Research (ICCIR) University Medical Center Hamburg-Eppendorf Hamburg Germany

[4]Department of Trauma, Hand and Reconstructive Surgery, University Medical Center Hamburg-Eppendorf, Hamburg, Germany

[§] The first two authors contributed equally to this manuscript.

*Corresponding author:

Dr. med. Dr. rer. nat. Felix N. von Brackel, M.Sc.

University Medical Center Hamburg-Eppendorf, Germany

Felix von Brackel: F.von-Brackel@uke.de

Mesh sensitivity analysis

Mesh sensitivity on naïve model

Initial sensitivity analysis was performed on the naïve femur model using a 2% criterion. Peak principal tensile stress on the distal segment of femur was used as the output parameter for the mesh convergence analysis. The initial mesh size was set 5 mm and the increment for alteration of the size was 0.5 mm. The results indicated convergence of results at the seeding size of 2 mm (see fig S1).

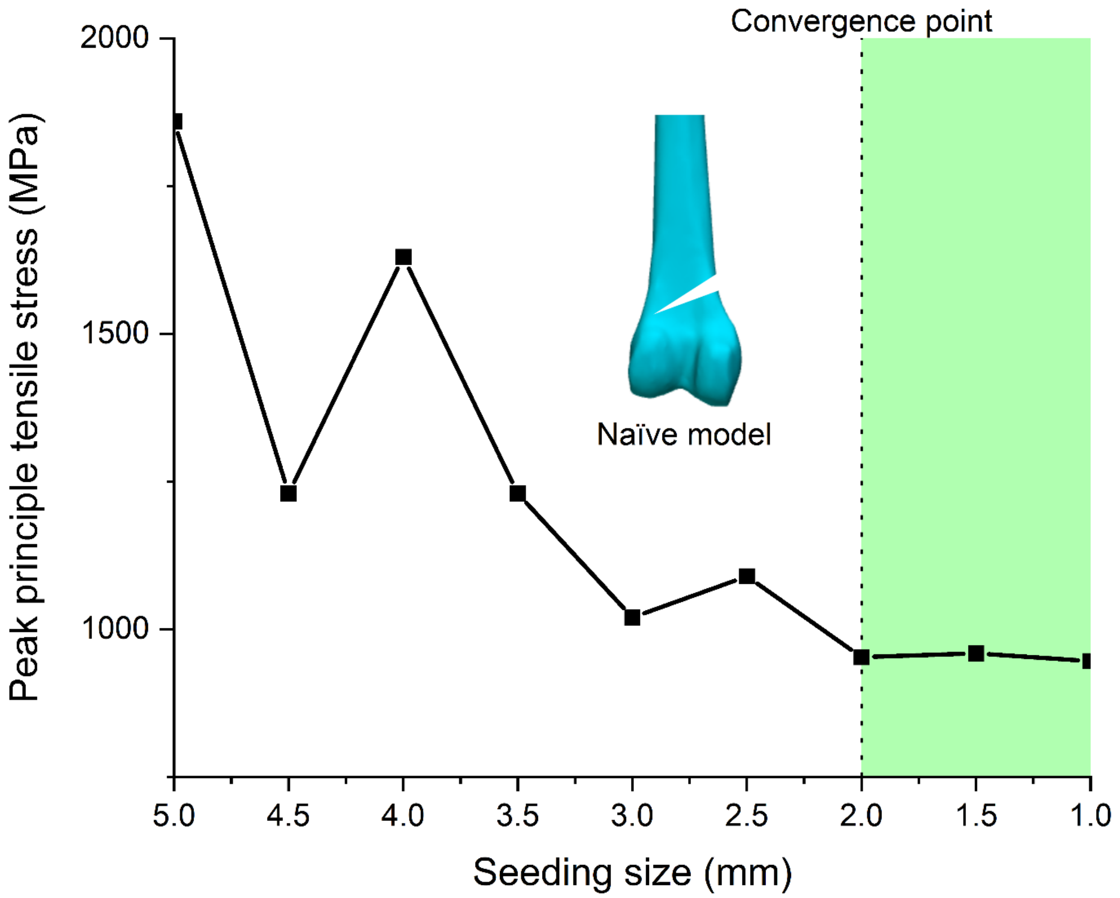

Figure S1. Convergence results based on peak principle tensile stress on the naïve model.

Mesh sensitivity on k-wire model

Secondary sensitivity analysis was performed on the A 40° - D 1.8 mm k-wire model using a 2% criterion. Peak principal tensile stress on the distal segment of femur was used as the output parameter for the mesh convergence analysis. The initial mesh size was set 0.35 mm which was the minimum element size that preserved the cross-sectional roundness of the k-

wire and the increment for alteration of the size was 0.05 mm. The results indicated convergence of results at the seeding size of 0.2 mm (see fig S2).

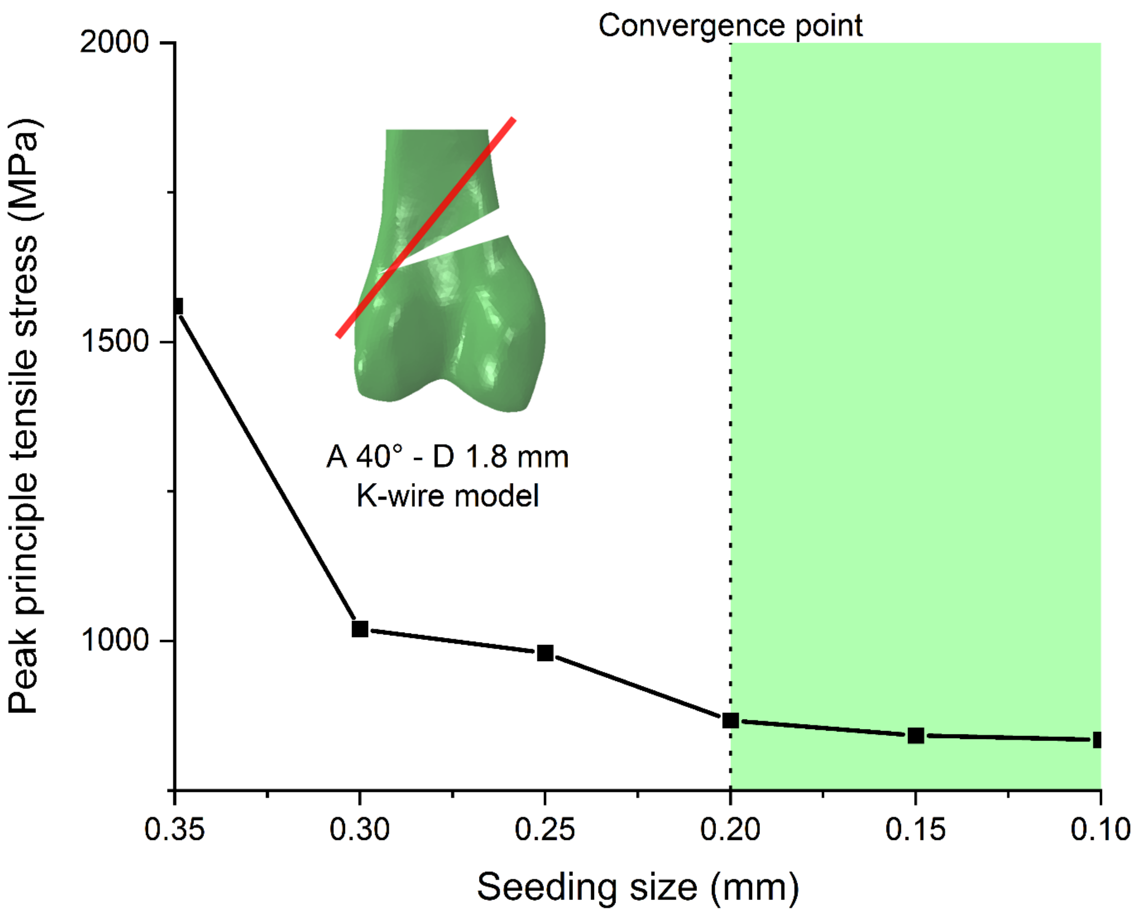

Figure S2. Convergence results based on peak principle tensile stress on the A 40° - D 1.8 mm k-wire model.

Statistical analysis

Table S1: 95% confidence interval of the multiple comparisons carried out using Bonferroni's test. Tens. Stresses=maximum tensile principal stresses; Comp. Stresses=maximum compression principal stresses; Vol. fraction=volumetric fraction of plastically deformed elements; CI=confidence interval; diff=difference

Variable	Bonferroni's Comparison	Tens. Stresses	Comp. Stresses	Vol. fraction	Stiffness
		95,00% CI of diff.			
Angle	30° vs. 45°	-1,156 to 539,8	-414,7 to 216,7	8,353 to 15,40	-1356 to 363,5
	45° vs. 60°	-239,5 to 301,5	-379,3 to 252,0	7,333 to 14,38	-728,1 to 991,8
	30° vs. 60°	29,84 to 570,8	-478,3 to 153,0	19,21 to 26,25	-1225 to 495,4
Diameter	1.6 vs. 1.8	-601,2 to 369,9	-129,9 to 369,9	-29,50 to 31,82	-561,5 to 1285
	1.6 vs. 2	-479,2 to 491,9	-347,5 to 152,2	-29,33 to 31,99	-972,6 to 874,4
	1.8 vs. 2	-363,5 to 607,5	-467,5 to 32,19	-30,49 to 30,83	-1335 to 512,4
